# Mutual regulation between chicken telomerase reverse transcriptase and the Wnt/β-catenin signalling pathway inhibits apoptosis and promotes the replication of ALV-J in LMH cells

**DOI:** 10.1186/s13567-021-00979-x

**Published:** 2021-08-19

**Authors:** Yong Xiang, Yun Yu, Qingbo Li, Zeng Jiang, Jinqun Li, Canxin Liang, Jian Chen, Yu Li, Xiaoyan Chen, Weisheng Cao

**Affiliations:** 1 College of Veterinary Medicine, South China Agricultural University, Guangzhou, 510642 China; 2 Key Laboratory of Zoonosis Prevention and Control of Guangdong Province, South China Agricultural University, Guangzhou, 510642 China; 3 Guangdong Laboratory for Lingnan Modern Agriculture, South China Agricultural University, Guangzhou, 510642 China; 4 National and Regional Joint Engineering Laboratory for Medicament of Zoonosis Prevention and Control, South China Agricultural University, Guangzhou, 510642 China; 5 Key Laboratory of Veterinary Vaccine Innovation of the Ministry of Agriculture, Guangzhou, 510642 China

**Keywords:** chicken telomerase reverse transcriptase, Wnt/β-catenin signalling pathway, cell proliferation, apoptosis, avian leukosis virus subgroup J

## Abstract

This study aimed to explore the mutual regulation between chicken telomerase reverse transcriptase (chTERT) and the Wnt/β-catenin signalling pathway and its effects on cell growth and avian leukosis virus subgroup J (ALV-J) replication in LMH cells. First, LMH cells stably overexpressing the chTERT gene (LMH-chTERT cells) and corresponding control cells (LMH-NC cells) were successfully constructed with a lentiviral vector expression system. The results showed that chTERT upregulated the expression of β-catenin, Cyclin D1, TCF4 and c-Myc. chTERT expression level and telomerase activity were increased when cells were treated with LiCl. When the cells were treated with ICG001 or IWP-2, the activity of the Wnt/β-catenin signalling pathway was significantly inhibited, and chTERT expression and telomerase activity were also inhibited. However, when the β-catenin gene was knocked down by small interfering RNA (siRNA), the changes in chTERT expression and telomerase activity were consistent with those in cells treated with ICG001 or IWP-2. These results indicated that chTERT and the Wnt/β-catenin signalling pathway can be mutually regulated. Subsequently, we found that chTERT not only shortened the cell cycle to promote proliferation but also inhibited apoptosis by downregulating the expression of Caspase 3, Caspase 9 and BAX; upregulating BCL-2 and BCL-X expression; and promoting autophagy. Moreover, chTERT significantly enhanced the migration ability of LMH cells, upregulated the protein and mRNA expression of ALV-J and increased the virus titre. ALV-J replication promoted chTERT expression and telomerase activity.

**Supplementary Information:**

The online version contains supplementary material available at 10.1186/s13567-021-00979-x.

## Introduction

Telomerase is a ribonucleoprotein enzyme that adds tandem (TTAGGG)n repeats to the ends of chromosomes in most eukaryotes [1]. The structure of chicken telomerase, which consists of chicken telomerase reverse transcriptase (chTERT), telomerase RNA component and telomerase-associated protein, is similar to that of human telomerase [2, 3]. Telomerase activation is considered a key step in cell carcinogenesis, and telomerase is one of the most commonly used molecular markers for a wide range of malignant tumours [4–6]. Studies have shown that human telomerase reverse transcriptase (hTERT) gene expression is the core determinant of telomerase activity, and overexpression of the hTERT gene is found in approximately 90% of human malignant tumours [7–9]. hTERT promoter activity is usually regulated by a variety of transcription factors (β-catenin, NF-κB and c-Myc), chromatin remodelling, and epigenetic methylation [10–16].

Avian leukosis is an infectious tumour caused by avian leukosis viruses (ALVs) belonging to the *Alpharetrovirus* genus of the family *Retroviridae* that affects poultry [17]. Chickens infected with exogenous ALV may suffer from decreased performance and immunosuppression, leading to tumour formation and even death [18, 19]. Avian leukosis is one of the most important diseases endangering the healthy development of poultry in China. Based on the specific envelope glycoprotein (gp85) expressed, ALV in naturally infected chicken flocks can be divided into seven subgroups (A, B, C, D, E, J and K). Endogenous subgroup E viruses with little to no pathogenicity are present in nearly all chicken lines, while the viruses of the other subgroups are exogenous and pathogenic. Subgroup A and B viruses mainly cause lymphocytic leukaemia and myeloid leukosis in field flocks, while flocks naturally infected with subgroup C and D viruses are rarely observed in the field. ALV subgroup J (ALV-J) is the most pathogenic to chickens, and it is more likely than viruses in the other ALV subgroups to cause tumours in chickens [20–22]. Yang et al. found ALV integration in the chTERT promoter/enhancer region in B cell lymphomas, suggesting that this is a common integration site, and chTERT expression was elevated by ALV integration [23]. Moreover, the chTERT gene was identified as a common insertion site in myeloid leukosis caused by ALV-J, and the gene expression of chTERT, a putative driver for oncogene activation, was significantly upregulated in tumour samples compared to control samples [24]. However, it is not clear whether chTERT affects ALV-J replication and what effect ALV-J has on chTERT.

The Wnt signalling pathway is a complex signalling pathway related to cell growth and development. Abnormal activation of Wnt is closely related to the development of cardiovascular disease, hepatic fibrosis and cancer [25, 26]. The Wnt/β-catenin signalling pathway, which is considered the most common Wnt signalling pathway, plays critical roles in various cellular functions, including cell proliferation, differentiation, migration and apoptosis [27–29]. The significance of this pathway is exemplified by evidence that both the overexpression and underexpression of target genes involved in this pathway result in various diseases. Appropriate regulation of the Wnt/β-catenin signalling pathway is key for cells to carry out their normal functions [28, 30]. The Wnt/β-catenin signalling pathway has been implicated in the transcriptional reactivation of hTERT in cancer cells; transient activation of Wnt/β-catenin induces hTERT mRNA expression and elevates telomerase activity in different cell lines [12, 31–33]. Blockade of Wnt/β-catenin signalling or regulation of chTERT activation may be effective therapeutic strategies for decreasing apoptotic events in cancer cells. However, whether Wnt/β-catenin impacts chTERT remains to be investigated. Therefore, in this study, in vitro models were used to assess chTERT expression and Wnt/β-catenin signalling in the LMH cell line and to explore the effects of chTERT and Wnt/β-catenin on cell proliferation, apoptosis, and migration and ALV-J replication to lay a theoretical foundation for further elucidating the mechanism of chTERT in the tumorigenic effect of ALV-J.

## Materials and methods

### Antibodies

Anti-β-catenin and anti-c-Myc primary antibodies were purchased from Santa Cruz Biotechnology (Santa Cruz, CA, USA). Anti-TCF4 and anti-Cyclin D1 primary antibodies were purchased from Affinity Biosciences, Ltd. (USA). An anti-GAPDH primary antibody was purchased from Abcam, Inc. (UK). An anti-HA tag antibody was purchased from Invitrogen, Thermo Fisher Scientific, Inc. (California, USA). An anti-Ki67 (chicken) antibody was purchased from GeneTex, Inc. (USA). A goat anti-rabbit IgG (FITC) secondary antibody and a goat anti-mouse IgG (FITC) secondary antibody were purchased from LI-COR Biosciences, Ltd. (Nebraska, USA). Mouse monoclonal antibodies (G2.3) against the gp85 protein of ALV-J were donated by Avian Disease and Oncology Laboratory (USA).

### Cell culture and viruses

The chicken hepatocellular carcinoma cell line LMH and the 293T cell line were kindly provided by Professor Ming Liao of South China Agricultural University. The chicken fibroblast cell line DF-1 was maintained in our laboratory. LMH, 293T and DF-1 cells were cultured in DMEM/F12 (Gibco, Thermo Fisher Scientific, Inc., USA) and DMEM (Gibco, Thermo Fisher Scientific, Inc.) supplemented with 10% foetal bovine serum (FBS) at 37 °C with 5% CO_2_. The ALV-J CHN06 strain was maintained in our laboratory.

### Construction of a chTERT gene expression plasmid and a stable overexpression cell line

The full-length chTERT coding region was amplified by RT-PCR from broiler embryos (4–7 days) as two overlapping fragments (chTERT-T1 and chTERT-T2) using Phanta® Max Super-Fidelity DNA Polymerase (Vazyme, Nanjing, China). Primers were designed according to relevant literature [34], and the sequences are shown in Table 1. The PCR product of chTERT-T2 was amplified again to add a linker and an HA tag to the end of the DNA to generate chTERT-T2-HA. The reaction conditions were as follows: 1 cycle of 95 °C for 5 min; 35 cycles of 95 °C for 45 s, 62 °C for 30 s and 72 °C for 150 s; and 1 cycle of 72 °C for 8 min. The annealing temperature was adjusted according to the Tm value of the primer. Since there is no commercially available antibody for chTERT, the HA tag was used to measure chTERT protein levels. Then, the chTERT-T1 and chTERT-T2-HA fragments were ligated into the pLV-sfGFP(2A) (puromycin) lentiviral expression vector at the Xba I and Xho I sites with a ClonExpress®Ultra One Step Cloning Kit (Vazyme, Nanjing, China) through homologous recombination to generate pLV-chTERT-HA. Lentiviral transfection and screening were performed according to the user manual for the lentiviral gene transfer and expression system (Inovogen, Wuhan, China). Briefly, the pLV-chTERT-HA plasmid was transfected into 293T cells using PolyJet™ In Vitro DNA transfection reagent (SignaGen, Maryland, USA). At 48 to 72 h post-transfection, the lentivirus in the supernatant was harvested, filtered through a 0.45-μm filter and used to infect LMH cells. After LMH cells were infected with lentivirus containing chTERT or empty control for 48 h, puromycin was added to kill uninfected cells. Finally, LMH cells stably overexpressing the chTERT gene (LMH-chTERT cells) or control (LMH-NC cells) were obtained.

**Table 1:** **Primers used for gene cloning and expression analysis**

Primer name^a^	Primer sequence (5’-3’)
chTERT-T1-F (Xba I)	CTCTACTAGAGGATCtctagaCGTGGGGCCCGCTGCACGGCAGCG^b^
chTERT-T1-R	ATACGCAGTCATTCACTCTCATCTTCCACATC
chTERT-T2-F	GCCATAACAAATGCCGGTTCTTTAAAAACGTG
chTERT-T2-R	GGGGTACCAGACCTTCATCCCTTAGTCCAG
chTERT-T2-linker-F	GTGATCCGCTCCTCAGGGAGCTGCTCAGGCAGCACAGCA GCCACTGGCAGGTGTATGGCTTTGTGAGGG
chTERT-T2-linker-R	CGATCCGCCACCGCCAGAGCCACCTCCGCCTGAACCGCC TCCACCGGTACCGTCCAGTATAGTTTTGAAAT
chTERT-T2-HA-F	GTGATCCGCTCCTCAGGGAGCTGCTCAGGCAGCACAGCA GCCACTGGCAGGTGTATGGCTTTGTGAGGG
chTERT-T2-HA-R (Xho I)	GGGCCCGGGTTCGAActcgagTTAAGCGTAGTCTGGGACGT CGTATGGGTATCGCGACGATCCGCCACCGCCAGAGCCAC^2^

^a^F: forward primer, R: reverse primer

^b^The lowercase letters indicate restriction sites

### Cell treatment

To inhibit the expression of Wnt/β-catenin and chTERT, cells were transfected with small interfering RNA (siRNA) targeting β-catenin (si-β-catenin, 5′-GCUGGUGGGAUGCAAGCUUTT-3′, 5′-AAGCUUGCAUCCCACCAGCTT-3′) or chTERT (si-chTERT, 5′-GCAUGGAACCUCCUGGCAUTT-3′, 5′-AUGCCAGGAGGUUCCAUGCTT-3’) or a nonspecific siRNA (si-NC, 5′-ACGUGACACGUUCGGAGAATT-3′, 5′-UUCUCCGAACGUGUCACGUTT-3′) (10 μmol/L) (Shanghai GenePharma Company). Cells plated in 12-well plates (100 000 cells/well) were transfected with siRNA using siRNA-Mate Transfection Reagent (GenePharma, Shanghai, China). In addition, ICG001 and IWP-2 (Beyotime, Shanghai, China) and LiCl (Sigma-Aldrich, St. Louis, Missouri, USA) were added directly to the cell culture medium to inhibit and activate Wnt/β-catenin signalling pathway activity, respectively [35–37]. After treatment for at least 24 h, cell viability and mRNA and protein expression were assessed by MTT assays, RT-PCR and Western blotting, respectively.

### MTT assay

Cytotoxicity was measured using the MTT assay with an Enhanced Cell Counting Kit-8 (Beyotime). The assay was performed according to the user manual. Briefly, LMH cells were plated in 96-well plates at a density of 1 × 10^4^ cells/well in 200 μL medium for 12 h and then treated with ICG001, IWP-2, LiCl and siRNA at different concentrations. After 72 h, CCK-8 reagent was added to the cells in the plate, the cells were incubated at 37 °C for 2 h, and the absorption was measured at 450 nm using a microplate reader (BioTek, Vermont, USA). Relative absorbance was calculated according to the following formula: cell viability = (OD_treated_ − OD_blank_)/(OD_untreated_ − OD_blank_), where OD is the optical density at 450 nm.

### Real-time fluorescence quantitative PCR (RT-PCR)

Total RNA was extracted from LMH cells by using TRIzol reagent (Fastagen Biotech, Shanghai, China) according to the manufacturer’s recommendations. First-strand cDNA was synthesized from 500 ng of total RNA template with random primers using a PrimeScript RT Reagent kit (TaKaRa, Japan). RT-PCR was performed using Hieff® qPCR SYBR Green Master Mix (YEASEN, Shanghai, China) on the CFX96™ Real-time PCR System (Bio-Rad, California, USA). Expression levels were quantified using the 2^−ΔΔCt^ method and normalized to GAPDH expression. All the primer sequences are shown in Table 2.

**Table 2:** **Primers used for real-time fluorescence quantitative PCR**

Gene	Primer sequence (5’-3’)	Reference
chTERT	F: GTAAGACTAAGCCGTGTTGTTG	[23]
	R: CTCCCGAATACTGAAGAGC	
β-catenin	F: GCCCTGCTCAACAAGACAAA	[54]
	R: CTGACAACACCTTCAGCACC	
Cyclin D1	F: TCGTTCGAACCCCTCAAGAA	[54]
	R: GCGGTCAGAGGAATCGTTTC	
TCF4	F: CCCTTACCCAACAGCTCTGA	[54]
	R: CTATGGCCGGATGAGGGATT	
c-Myc	F: AGCGACTCGGAAGAAGAACAAG	[55]
	R: ATCGACTTCGCTTGCTCAGACT	
BCL-2	F: ATCGTCGCCTTCTTCGAGTT	[49]
	R: ATCCCATCCTCCGTTGTCCT	
BAX	F: TGGATTCTCACAGTAGGAGGATG	[49]
	R: CGTAGACCTTGCGGATAAAGC	
BCL-X	F: CTTTCAGCGACCTCACCTC	[49]
	R: ACAATGCGTCCCACCAGT	
Caspase 9	F: CCGAAGGAGCAAGCACG	[56]
	R: AGGTTGGACTGGGATGGAC	
Caspase 3	F: CATCTGCATCCGTGCCTGA	[56]
	R: CTCTCGGCTGTGGTGGTGAA	
ALV-J	F: TGTGTGCGTGGTTATTATTTC	[57]
	R: AATGGCGAGGTCGCTGACTGC	
GAPDH	F: GGAAAGTCATCCCTGAGCTG	[23]
	R: GGTCAACAACAGAGACATTGG	

### Western blotting

Protein was extracted from LMH cells using Fierce RIPA Lysis Buffer (Beyotime) following the manufacturer’s instructions. Briefly, equal amounts of total protein were separated by SDS–polyacrylamide gel electrophoresis (SDS-PAGE) (Beyotime) and then transferred onto a polyvinylidene difluoride (PVDF) membrane. The membranes were immunoblotted with primary antibodies at 4 °C overnight, followed by incubation with a fluorescein isothiocyanate-conjugated secondary antibody at 37 ℃ for one hour. Finally, the blots were scanned using an Odyssey Infrared Imaging System (LI-COR, Nebraska, USA).

### Telomerase activity assay

Telomerase activity was measured by the telomeric repeat amplification protocol (TRAP), which was conducted with the TeloTAGGG Telomerase PCR ELISA Kit (Sigma-Aldrich) following the manufacturer’s protocol. A total of 2 × 10^5^ cells were transferred to an Eppendorf tube and centrifuged. The pellet was resuspended in lysis buffer (200 μL) and incubated for 30 min on ice, and then, the supernatant was collected and transferred to a fresh tube. A total of 25 μL reaction mixture was transferred to an amplification tube, and 23 μL PCR-grade water and 2 μL sample or positive (293T cell lysate) or negative (DF-1 cell lysate) control were added to obtain a 50-μL PCR system. Amplification was performed on a PCR instrument (Applied Biosystems, Foster City, CA, USA) under the following conditions: 1 cycle of 25 ℃ for 20 min; 1 cycle of 94 ℃ for 5 min; 30 cycles of 94 ℃ for 30 s, 50 ℃ for 30 s and 72 °C for 90 s; and 1 cycle of 72 ℃ for 10 min. Then, 20 μL denaturation reagent and 5 μL amplification products were added sequentially to the tube, and the mixture was incubated for 10 min at room temperature. Hybridization buffer (225 μL) was added, followed by thorough mixing, and 100 μL of the mixture was transferred to each microplate well and incubated for 2 h at 37 °C. After 3 washes, anti-DIG-POD working solution (100 μL) was added, and the samples were incubated for 30 min at room temperature. Then, after 5 washes, TMB substrate solution (100 μL) was added and incubated for 15 min at room temperature. Finally, 100 μL top reagent was added, and the absorbance at 450 nm was measured within 15 min.

### Flow cytometry

Cell proliferation, apoptosis and cell cycle were analysed by flow cytometry using the BeyoClick™ EdU Cell Proliferation Kit with Alexa Fluor 647 (Beyotime), Annexin V-APC Apoptosis Analysis Kit (Sungene Biotech, Tianjin, China) and Cell Cycle Kit (Beyotime), respectively, according to the manufacturer’s protocol. Cells were acquired on a CytoFLEX flow cytometer (Beckman Coulter, Florida, USA), and data were analysed by FlowJo software (Tree Star, Inc., Ashland, OR, USA).

### Immunofluorescence analysis

Apoptosis was analysed by immunofluorescence using a One Step TUNEL Apoptosis Assay Kit (Beyotime) according to the manufacturer’s protocol. Cell proliferation was also analysed by Ki67 immunofluorescence. Seventy-two hours after LMH-chTERT and LMH-NC cells were plated in 6-well plates at a density of 1 × 10^5^ cells/well, the cells were fixed with 3% formaldehyde and 2% sucrose in PBSA and then permeabilized with 0.1 M glycine. The cells were blocked with 2% normal goat serum and 0.4% Triton X-100 in PBSA for 30 min and incubated overnight with primary antibody. After being washed with 0.2% Triton X-100 in PBSA, the cells were incubated with Cy3-labelled goat anti-rabbit IgG secondary antibody, and DAPI was used for nuclear visualization. Images were captured using a Nikon Ti2 microscope and Nikon camera.

### Transmission electron microscopy (TEM)

LMH cells were plated into 6-well cell culture plates, incubated for 24 h, harvested, and fixed in 2.5% glutaraldehyde for 24 h at 4 °C. Subsequently, the cells were rinsed with buffer, fixed with citric acid, and dehydrated in a series of ethanol solutions. Then, the cells were permeabilized in different ratios of ethanol: resin and embedded in pure resin, which was polymerized at 70 °C. The stained cells were visualized using a transmission electron microscope (Hitachi, Ltd., Japan) to observe various structures within the cells.

### Wound healing assay

Cell culture inserts (Ibidi, Martinsried, Germany) were placed in 6-well plates, and LMH cells were plated on the inserts. After the cell density reached 100%, the inserts were removed, a wound was created, the supernatant was discarded, and Opti-MEM (Gibco, California, USA) without serum was added to culture the cells. After 24 and 48 h, the cells were imaged with a Nikon Eclipse Ti2 microscope, and the pixel area of the wound gap was calculated using ImageJ v1.49 (NIH, USA). The migration rate (MR) was calculated as (initial pixel area (0 h)-terminal pixel area (24 h or 48 h))/initial pixel area (0 h) × 100%.

### Enzyme-linked immunosorbent assay (ELISA) for ALV p27

Cells infected with ALV-J CHN06 at a multiplicity of infection (MOI) of approximately 0.1 were incubated at 37 °C with 5% CO_2_ for 7 days. Then, ALV group-specific p27 antigen levels in the cell culture supernatant were measured with an Avian Leukosis Virus Antigen Test Kit (IDEXX Laboratories Pty., Ltd., Westbrook, USA) according to the manufacturer’s protocol. Briefly, samples and positive and negative controls (100 μL) were added to the wells of ELISA plates, incubated at room temperature for 60 min, and then washed three to five times with 350 μL/well washing buffer. Then, 100 μL HRP-conjugated rabbit antibody against p27 was added to all the wells, and the plate was incubated for another 60 min. After 3–5 washes, 100 μL substrate solution was added for colour development for 15 min, and the reaction was stopped by adding 100 μL stop solution to each well. The absorbance at 650 nm was measured using a microplate reader (BioTek). The expression level of ALV p27 was determined by calculating the S/P value: [(mean sample OD) − (mean negative control OD)]/[(mean positive control OD) − (mean negative control OD)].

### TCID_50_ assay

The TCID_50_ assay was used to evaluate the virus titre. DF-1 cells were cultured in 96-well plates at a density of 4 × 10^4^ cells per well. A tenfold serially diluted virus sample was added to each well in a volume of 100 μL (eight replicates for each sample) and incubated for 2 h. Then, the supernatant was discarded, fresh DMEM containing 1% FBS was added, and the cells were incubated for 7 continuous days at 37 °C in 5% CO_2_. ELISAs for ALV p27 were performed. The TCID_50_ was calculated using the Reed-Muench method.

### Statistical analysis

All the results are presented as the mean ± standard deviation. Statistical analysis was performed by Student’s *t* test using GraphPad 5 software. A *p* value of < 0.05 was considered significant. *, **, *** and **** indicate *p* values less than 0.05, 0.01, 0.001 and 0.0001, respectively.

## Results

### Cloning of the chTERT gene

The chTERT gene, which is approximately 4000 bp in length, was obtained by PCR as described in the Methods. The results of agarose gel electrophoresis of the amplification products for the chTERT-T1, chTERT-T2 and chTERT-T2-HA fragments and the double restriction digestion of the pLV-chTERT-HA plasmid are shown in Additional file 1. The recombinant plasmids were sent to Sangon Biological (Shanghai, China) for sequencing, and the results showed that the full-length coding sequence of chTERT was 4097 bp long and was predicted to encode a protein of 1346 amino acids (aa). The aa sequence of the protein was nearly identical to that of the *Gallus gallus* TERT sequence [GenBank: NM_001031007.1] except for one transition mutation (a V-I mutation at aa 326 and an R-H mutation at aa 384).

### Stable overexpression of the chTERT gene in LMH cells

**Figure 1:**
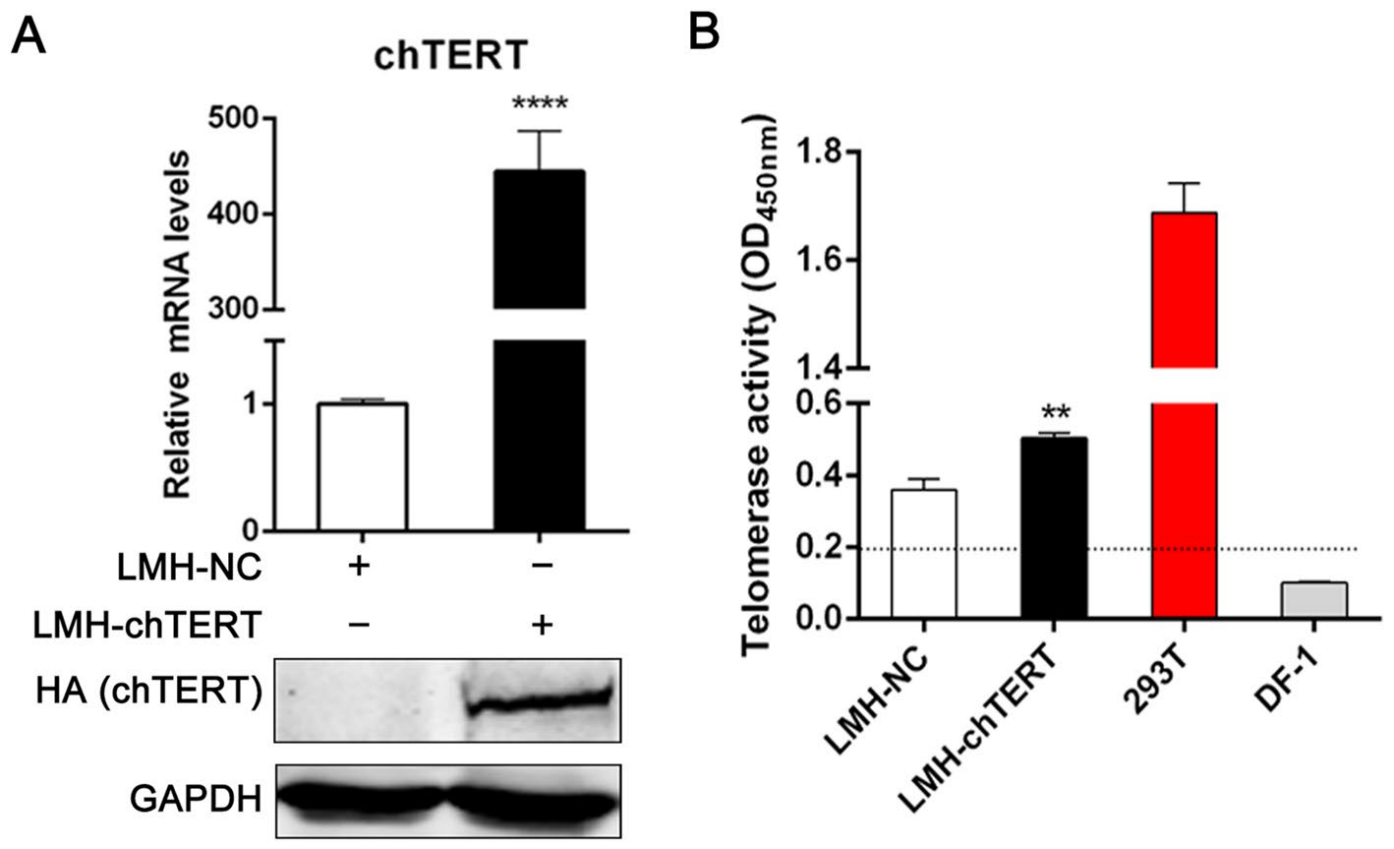
**Stable overexpression of the chTERT gene in LMH cells**. **A** The mRNA and protein levels of chTERT. **B** Analysis of telomerase activity. Cells with an OD_450nm_ greater than or equal to 0.2 were considered telomerase positive.

The pLV-chTERT-HA and pLV-NC plasmids were used to infect LMH cells. After repeated screening with puromycin, LMH cells stably overexpressing the chTERT gene (LMH-chTERT cells) and negative control cells (LMH-NC cells) were obtained. The results showed that the mRNA and protein expression levels of chTERT were significantly higher in LMH-chTERT cells than in control cells (Figure 1A; *p* < 0.0001). Although LMH cells were telomerase positive, the expression level of chTERT was extremely low. In addition, telomerase activity in LMH-chTERT cells was significantly higher than that in control cells (Figure 1B; *p* < 0.01) but lower than that in 293 T cells, which were regarded as positive controls. DF-1 cells, which do not exhibit telomerase activity, were used as negative controls.

### Cytotoxic effects of LiCl, ICG001, IWP-2 and siRNA on LMH cells

**Figure 2:**
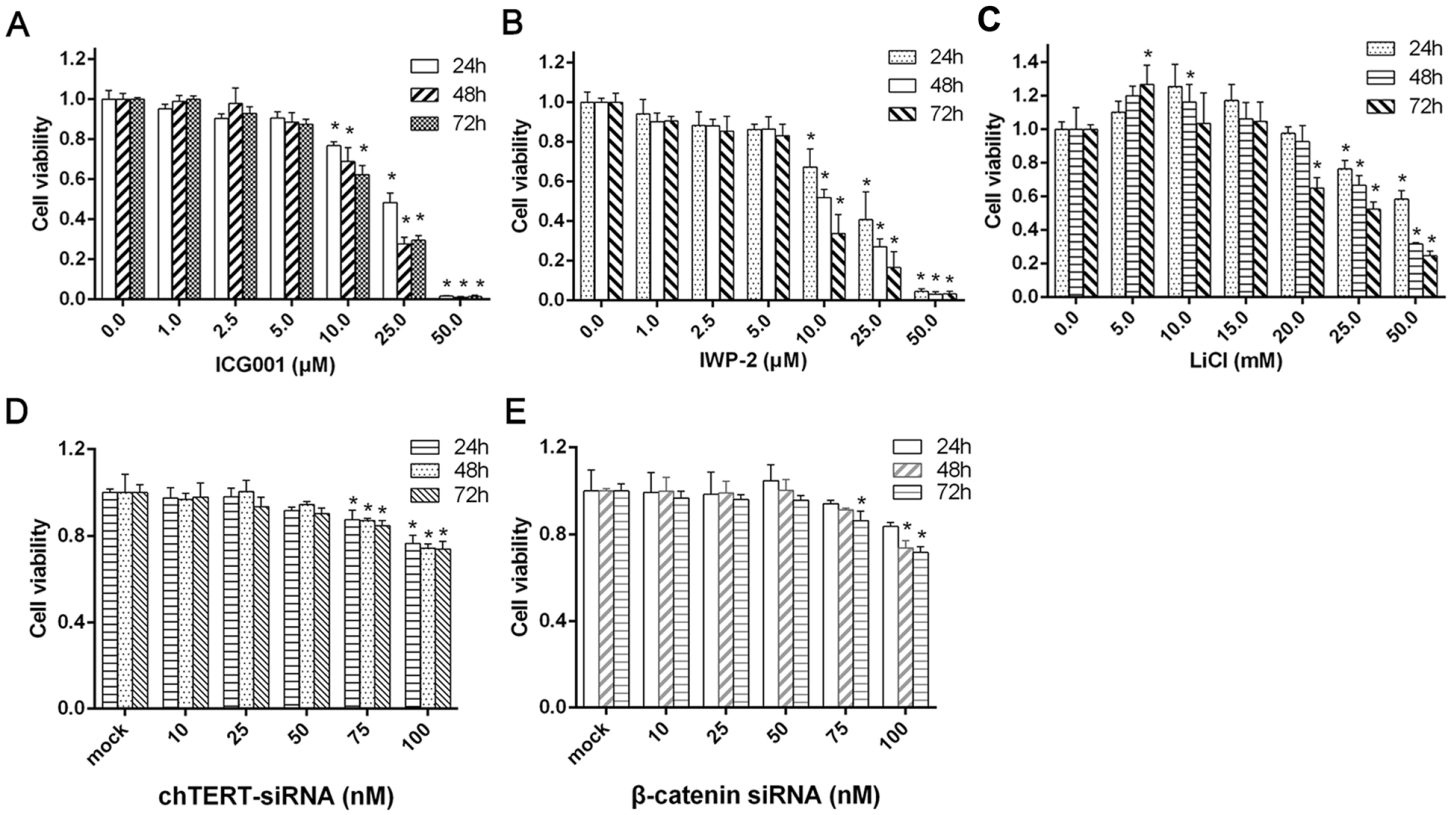
**MTT assay**. Cytotoxic effects of ICG001 (**A**), IWP-2 (**B**), LiCl (**C**), si-chTERT (**D**) and si-β-catenin (**E**) on LMH cells.

To determine the concentration range at which the investigated inhibitors and siRNAs suppress β-catenin-dependent gene or chTERT gene expression without markedly affecting cell viability, the cytotoxic effects of Wnt/β-catenin signalling pathway inhibitors (ICG001 and IWP-2) and siRNAs (si-β-catenin and si-chTERT) were assessed by MTT assays. The cytotoxicity of a Wnt/β-catenin signalling pathway activator (LiCl) was also tested. For functional testing, the cells were incubated with an inhibitor or activator at different concentrations for 24, 36, or 72 h. Similarly, LMH cells were transfected with different concentrations of siRNA for 24, 36, or 72 h. Over a wide range of concentrations, ICG001, IWP-2, LiCl and siRNAs decreased cell viability in a dose-dependent manner. The reduction in LMH cell viability was not significantly different from that observed in the control group when ICG001 was administered at concentrations ≤ 5 μM, time had no obvious influence on cell viability at these concentrations (Figure 2A), and similar results were obtained with IWP-2 (Figure 2B). At a concentration of ≥ 20.0 μM, LiCl significantly decreased the viability of LMH cells; however, at 5.0 and 10.0 mM, LiCl significantly increased cell viability, and the effect of 5 mM LiCl was stronger than that of 10 mM LiCl (Figure 2C). In addition, both β-catenin siRNA and chTERT siRNA exhibited significant cytotoxicity at concentrations ≥ 75 nM (Figures 2D and E). Therefore, the optimal concentrations of ICG001, IWP-2, LiCl and siRNAs were 5 μM, 5 μM, 5 mM and 50 nM, respectively; these concentrations were used in subsequent experiments.

### chTERT activated the Wnt/β-catenin signalling pathway overexpression

**Figure 3:**
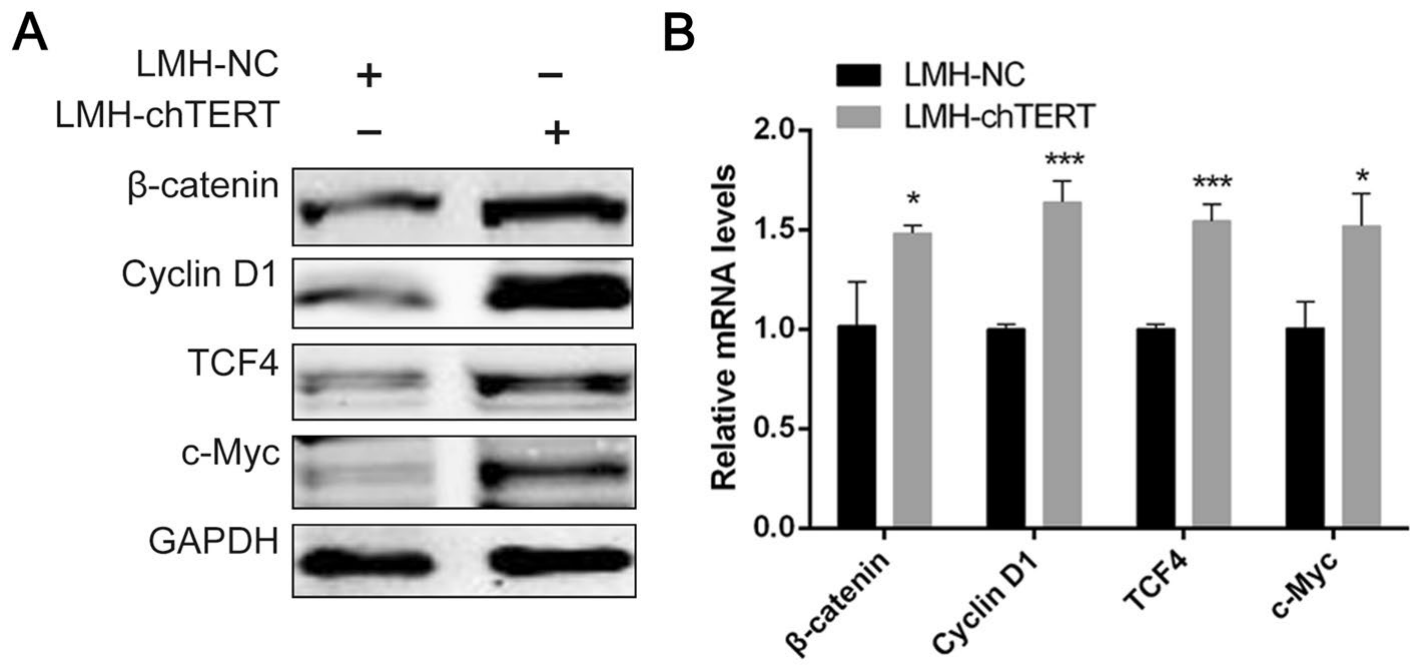
**chTERT activated the Wnt/β-catenin signalling pathway**. Western blotting (**A**) and RT-PCR (**B**) were used to measure the protein and mRNA expression levels of chTERT, respectively, in LMH-chTERT and LMH-NC cells.

The Western blot and RT-PCR results showed that chTERT significantly increased β-catenin protein expression, which was higher in LMH-chTERT cells than in LMH-NC cells. The overexpression of chTERT also significantly increased the expression of downstream target genes, such as cyclin D1, the transcription factor TCF4 and the tumour-related factor c-Myc (Figures 3A and B; *p* < 0.05). These results indicated that chTERT can activate the Wnt/β-catenin signalling pathway and affect cell growth.

### The β-catenin signalling pathway regulated chTERT expression and telomerase activity

**Figure 4:**
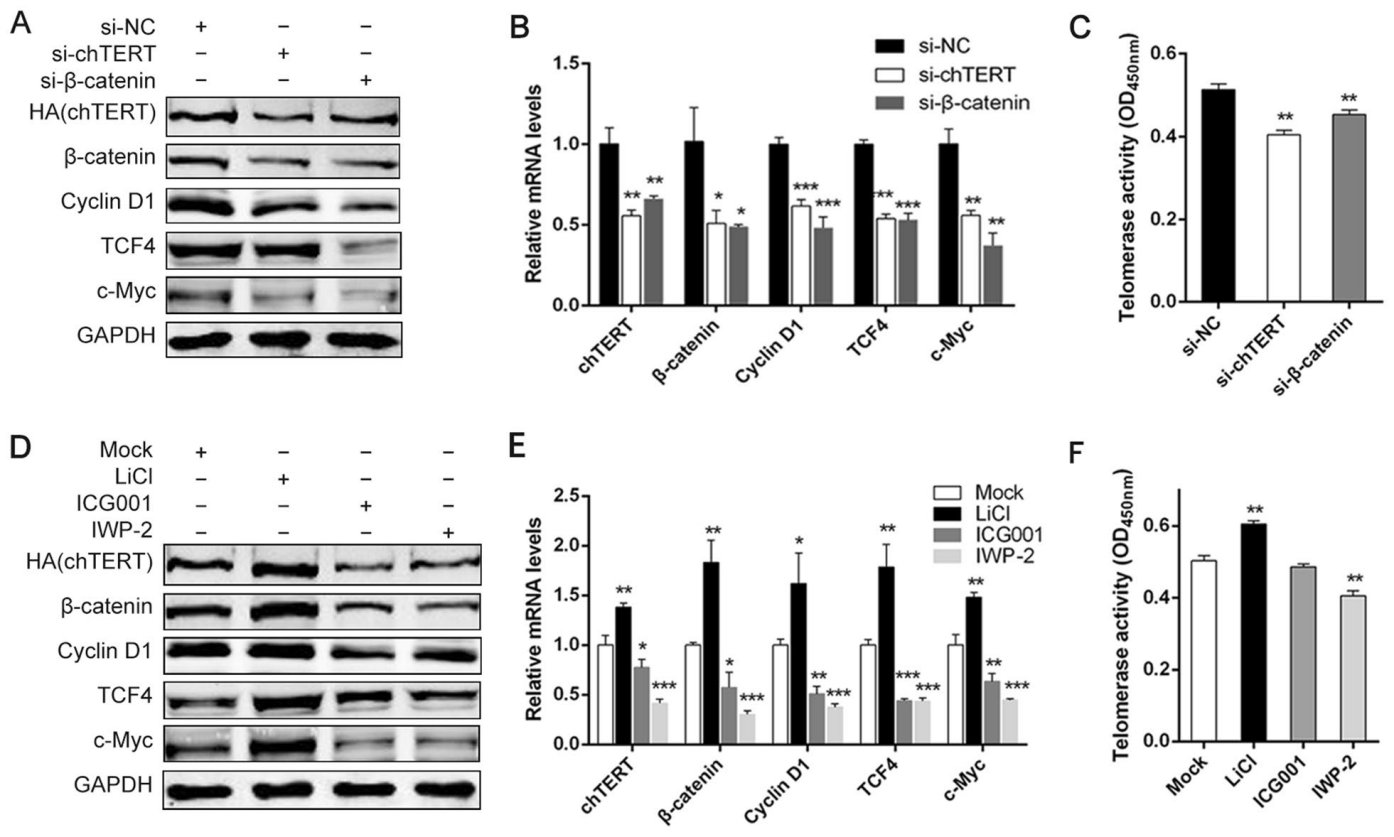
**Wnt/β-catenin and chTERT mutually regulated and promoted the activity/expression of each other.** Western blotting (**A**) and RT-PCR (**B**) were performed to assess the changes in chTERT expression and the Wnt/β-catenin signalling pathway in LMH-chTERT cells 72 h after siRNA transfection. **C** Measurement of telomerase activity in LMH-chTERT cells 72 h after transfection of si-chTERT or si-β-catenin. Western blotting (**D**) and RT-PCR (**E**) were performed to evaluate the changes in chTERT expression and the β-catenin signalling pathway in LMH-chTERT cells 72 h after treatment with inhibitors (ICG001 and IWP-2) or an activator (LiCl). **F** Measurement of telomerase activity in LMH-chTERT cells 72 h after treatment with LiCl, ICG001 and IWP-2.

To further verify the regulatory effect of chTERT on the Wnt/β-catenin signalling pathway, si-chTERT was used to interfere with chTERT expression in LMH-chTERT cells, and Wnt/β-catenin signalling pathway activity was determined at 72 h. In addition, to explore whether the Wnt/β-catenin signalling pathway can regulate the expression of chTERT, LMH-chTERT cells were treated with inhibitors (ICG001 and IWP-2), an activator (LiCl) or si-β-catenin for 72 h, and then, changes in chTERT expression and telomerase activity were measured by Western blotting, RT-PCR and TRAP. The results showed that when chTERT expression was inhibited by si-chTERT, β-catenin protein and mRNA expression was also inhibited, indicating that the activity of the Wnt/β-catenin signalling pathway was reduced (Figures 4A and B; *p* < 0.05) and that the activity of telomerase was inhibited (Figure 4C; *p* < 0.01). Further experiments verified that chTERT overexpression could increase the activity of the Wnt/β-catenin signalling pathway. Moreover, when cells were treated with ICG001, IWP-2 and β-catenin siRNA, the activity of the Wnt/β-catenin signalling pathway was inhibited, and the expression of chTERT was also significantly reduced (Figures 4A, D and E; *p* < 0.05). Similarly, telomerase activity in LMH-chTERT cells was reduced after treatment with IWP-2 (Figure 4F; *p* < 0.01). In contrast, the activity of the Wnt/β-catenin signalling pathway and the expression of chTERT were both increased after LiCl treatment (Figures 4D and E; *p* < 0.05), and telomerase activity was also significantly increased (Figure 4F; *p* < 0.01). These findings further suggested that the β-catenin signalling pathway and chTERT gene can mutually regulate each other and promote the activity/expression of the other.

### chTERT promoted the proliferation of LMH cells

**Figure 5:**
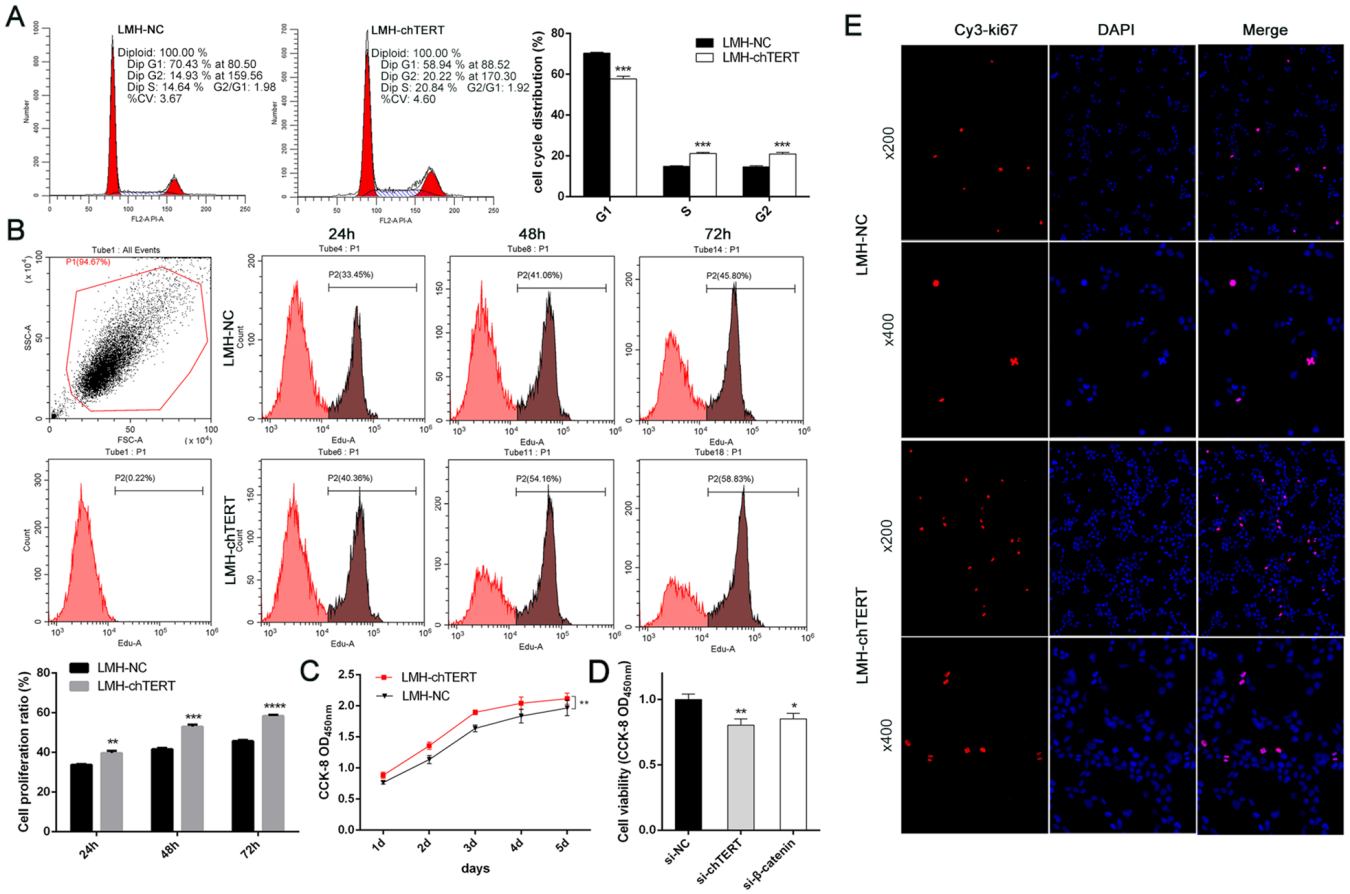
**chTERT promoted the proliferation of LMH cells.A** The cell cycle was analysed by flow cytometry. G1, the prophase of DNA synthesis. S phase, the DNA synthesis phase. G2, late phase of DNA synthesis. **B** Cell proliferation at 24, 48 and 72 h was analysed by flow cytometry. **C** The MTT assay was used to analyse the viability of LMH-chTERT and LMH-NC cells over 1–5 days. Growth curves were drawn after the values were read at a wavelength of 450 nm. **D** The MTT assay was used to analyse the viability of LMH-chTERT cells 72 h after transfection with siRNA. **E** Immunofluorescence staining of Ki67, an antigen associated with proliferating cells. Ki67 is closely related to mitosis and is indispensable for cell proliferation.

As the above results showed that chTERT increases the activity of the Wnt/β-catenin signalling pathway and promotes the expression of Cyclin D1, we postulated that chTERT affects the cell cycle and cell proliferation. To verify this conjecture, the cell cycle was analysed in LMH-chTERT and LMH-NC cells by flow cytometry. The results showed that chTERT overexpression promoted the transition of LMH cells from G1 to S phase and G2 and increased the ratio of S phase cells to G2 cells (Figure 5A; *p* < 0.001), indicating a reduction in the time required for cell replication, which could theoretically promote cell proliferation. Subsequently, flow cytometry was used to analyse cell proliferation. The results showed that overexpression of chTERT significantly promoted the proliferation of LMH cells (Figure 5B; *p* < 0.01). The same conclusion was obtained from growth curves for the two kinds of cells in MTT assays (Figures 5C and D; *p* < 0.05). Finally, Ki67 immunofluorescence staining verified that chTERT could promote the proliferation of LMH cells (Figure 5E).

### chTERT inhibited the apoptosis of LMH cells and induced a switch to autophagy

**Figure 6:**
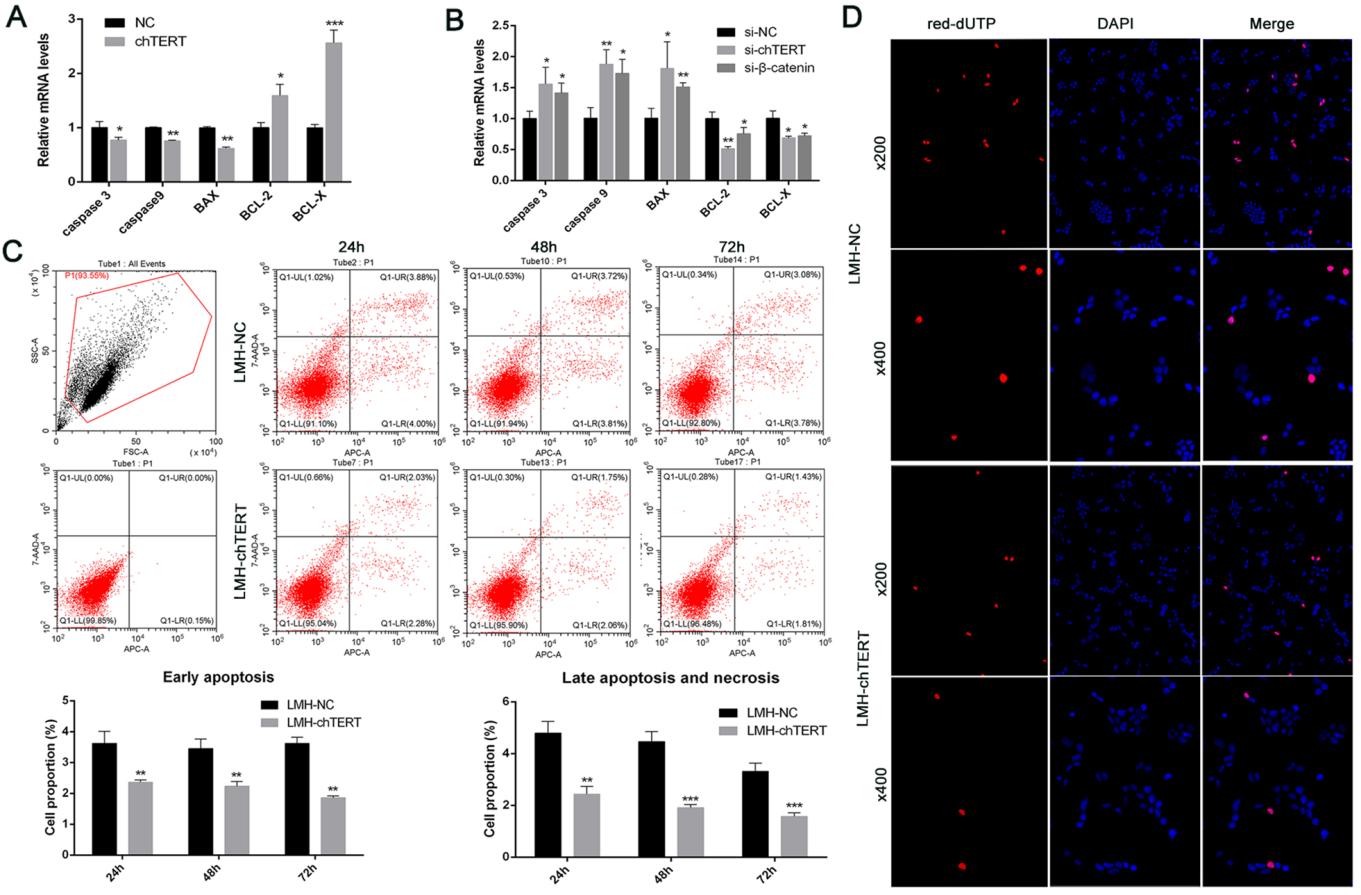
**chTERT inhibited the apoptosis of LMH cells**. **A**, **B** The mRNA levels of genes that promote apoptosis (Caspase 3, Caspase 9, and BAX) or inhibit apoptosis (BCL-2 and BCL-X) were measured by RT-PCR. **C** Flow cytometry analysis of early apoptotic, late apoptotic and necrotic LMH cells. APC-positive and 7-AAD-negative cells represent early apoptotic cells, and 7-AAD-positive cells represent late apoptotic or necrotic cells. **D** TUNEL staining was used to analyse late apoptotic LMH cells. In late apoptotic cells, chromosomal DNA breaks result in the production of a large number of sticky 3'-OH ends. Under the action of deoxyribonucleotide terminal transferase (TdT), deoxyribonucleotides, fluorescein, peroxide derivatives, alkaline phosphatase, or biotin are added to the 3'-end of the DNA, allowing apoptotic cells to be detected.

**Figure 7:**
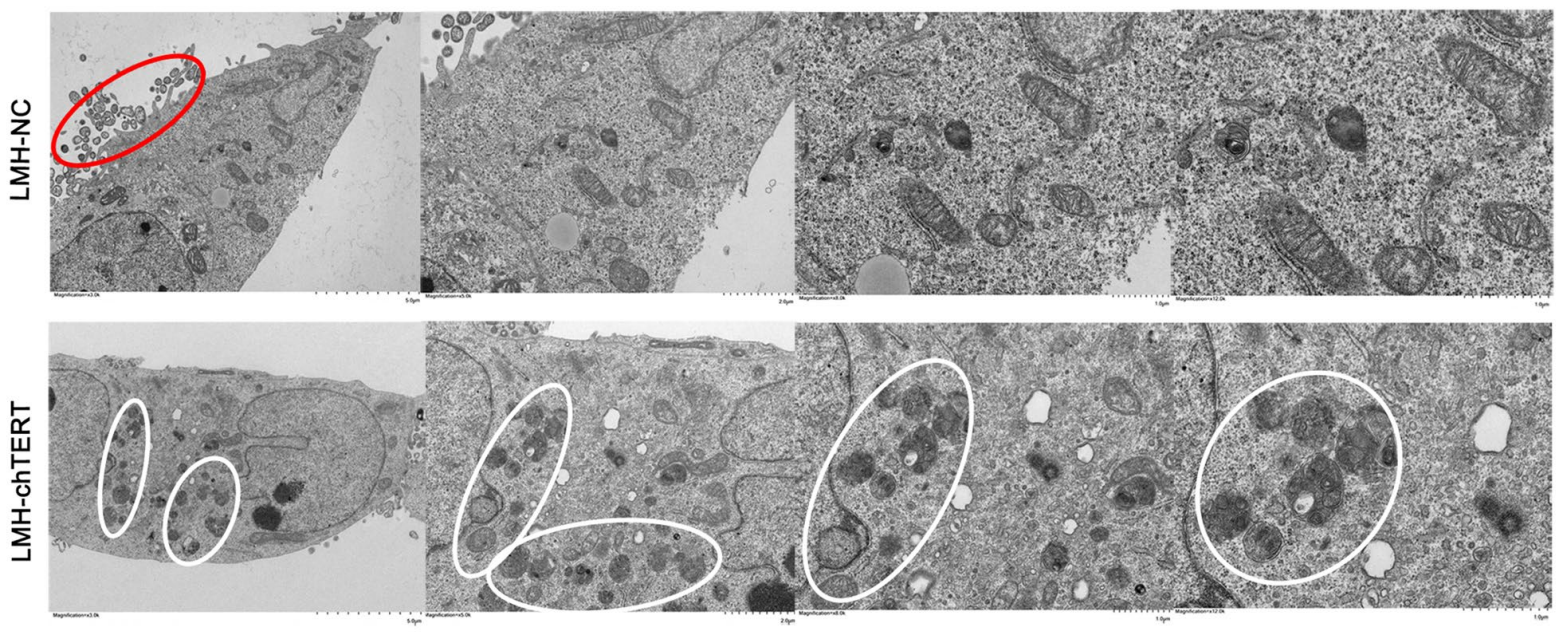
**Transmission electron microscopy**. chTERT inhibited the apoptosis of LMH cells and promoted the switch to autophagy. Apoptotic bodies are circled in red, and autophagosomes are circled in white.

Since regulation of the Wnt/β-catenin signalling pathway by the chTERT gene is often associated with cell growth and development, the effect of chTERT on the apoptosis of LMH cells was explored. The results showed that the mRNA levels of genes that promote apoptosis, such as Caspase 3, Caspase 9 and BAX, were significantly lower in LMH-chTERT cells than in control cells, while the mRNA levels of genes that inhibit apoptosis, such as BCL-2 and BCL-X, were significantly higher than in control cells (Figure 6A; *p* < 0.05). When the expression of the chTERT or β-catenin gene in LMH-chTERT cells was decreased by si-chTERT or si-β-catenin, similar results were obtained (Figure 6B; *p* < 0.05). The apoptosis of LMH cells was assessed in detail by flow cytometry, and the results showed that the proportions of early apoptotic, late apoptotic, and necrotic cells were lower in the LMH-chTERT group than in the LMH-NC group (Figure 6C; *p* < 0.01). In addition, the late apoptosis of LMH cells was analysed by terminal-deoxynucleotidyl transferase-mediated nick end labelling (TUNEL) staining. The proportion of TUNEL-positive cells was lower in the chTERT overexpression group than in the control group (Figure 6D). To verify this finding more intuitively, TEM was performed, and the results showed fewer apoptotic bodies in LMH-chTERT cells than in LMH-NC cells (Figure 7). It is worth noting that there were more autophagosomes in LMH-chTERT cells than in control cells. In addition to indicating that the chTERT gene can inhibit the apoptosis of LMH cells, this finding suggested that overexpression of the chTERT gene promotes the autophagy of LMH cells. These data suggest that chTERT can reduce apoptosis by inducing a switch to autophagy.

### chTERT promoted the migration of LMH cells

**Figure 8:**
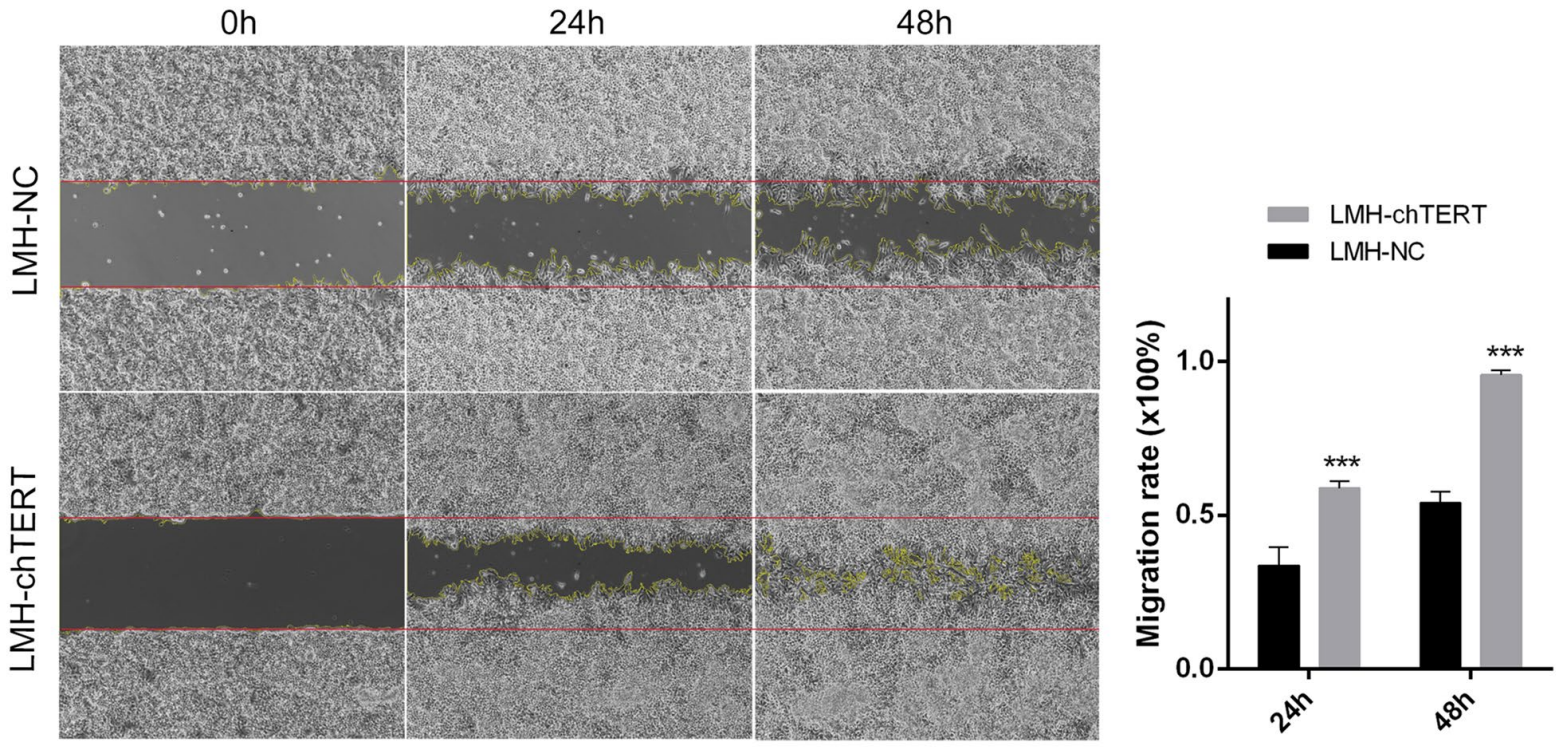
**chTERT promoted the migration of LMH cells.** When the cell density reached 100%, the original medium was discarded and replaced with serum-free medium for 48 h. Images of cells at the same location were taken at 0, 24, and 48 h. ImageJ software was used to calculate the pixel area of the wound healing region and assess cell mobility.

Our previous study found that chTERT is highly expressed in myeloid tumours caused by ALV-J, which may be related to tumour development and progression [24]. The effect of chTERT on LMH cell migration was analysed in vitro by the wound healing assay. The migration rate of LMH-chTERT cells was significantly higher than that of LMH-NC cells at 24 and 48 h (Figure 8; *p* < 0.001), indicating that chTERT gene expression enhances the migration ability of LMH cells and indirectly suggesting that chTERT can indeed promote the development and progression of tumours.

### chTERT promoted the replication of ALV-J in LMH cells

**Figure 9:**
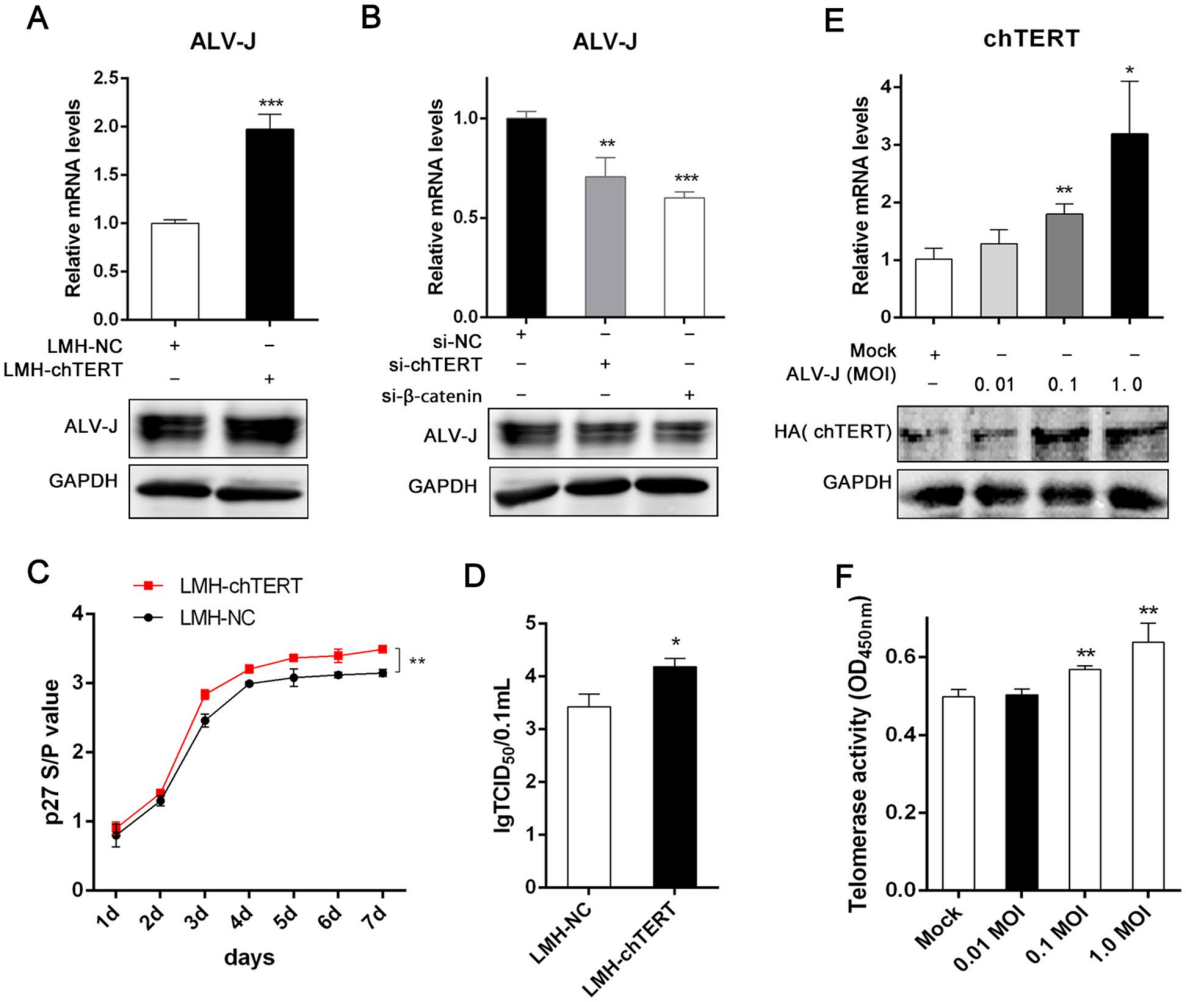
**chTERT promoted the replication of ALV-J in LMH cells.A** Seventy-two hours after LMH-chTERT and LMH-NC cells were infected with the same amount of ALV-J (CHN06), the level of ALV-J in the cell pellet was measured by RT-PCR and Western blotting. **B** LMH-chTERT cells were transfected with chTERT and β-catenin siRNAs and then inoculated with the same amount of ALV-J. After 72 h of incubation, the ALV-J level was measured by RT-PCR and Western blotting. The figure shows that after inhibiting chTERT gene expression or β-catenin signalling pathway activity, the replication of ALV-J was inhibited. **C** LMH-chTERT and LMH-NC cells were infected with the same amount of ALV-J virus. After 2 h of incubation, the virus solution was discarded and replaced with new culture medium containing 1% FBS for 7 days. Then, the supernatant was collected every day to detect the p27 antigen. **D** The effect of chTERT on the virus titre of ALV-J. **E** LMH-chTERT cells were infected with ALV-J at an MOI of 0.01, 0.1 or 1.0. After 72 h of incubation, the expression of chTERT in the cell pellets of the infected and uninfected groups was measured by RT-PCR and Western blotting, and telomerase activity was also assessed (**F**).

Since the chTERT gene is highly abundant in myeloid tumours caused by ALV-J, we asked whether it affects ALV-J replication in LMH cells. To answer this question, we assessed the change in chTERT expression in LMH-chTERT and LMH-NC cell pellets before and after infection with ALV-J for 72 h. The cell culture supernatant was collected every day, ALV p27 antigen levels were measured by ELISA, and a growth curve was drawn for ALV-J. The results showed that the expression level of ALV-J was significantly higher in LMH-chTERT cells than in control cells regardless of gp85 mRNA or protein level (Figures 9A and B; *p* < 0.01). Moreover, the content of ALV-J p27 antigen in the supernatant of LMH-chTERT cells was significantly higher than that in the supernatant of control cells (Figure 9C; *p* < 0.01), which was consistent with the TCID_50_ values (Figure 9D; *p* < 0.05). The above results indicated that chTERT can promote the replication of ALV-J in LMH cells. To explore whether ALV-J can affect the expression of chTERT, LMH-chTERT cells were infected with ALV-J at different MOIs, and an uninfected group was used as a control. The results showed that chTERT expression was significantly higher in cells infected with ALV-J at an MOI of 0.1 or 1.0 than in control cells (Figure 9E and F; *p* < 0.05) and that telomerase activity was also increased in infected cells, indicating that ALV-J can promote the expression of chTERT. These data indicate that chTERT probably plays an important role in the pathogenesis of ALV-J infection.

## Discussion

Telomerase reverse transcriptase is a key component of telomerase, which plays an important role in maintaining telomere stability and regulating cell growth and development. Telomerase reverse transcriptase has become an important target for the diagnosis and treatment of human tumours [38, 39]. Activation of the hTERT gene is strictly regulated by a variety of signalling pathways in different human tumours, specifically the Wnt/β-catenin, NF-κB and c-Myc signalling pathways [10, 13, 28, 40, 41]. However, during ALV-J-induced tumorigenesis, the molecular mechanism by which the chTERT gene regulates the Wnt/β-catenin signalling pathway and participates in cell growth and virus replication is still unknown. Therefore, exploring the interaction between the chTERT gene and the Wnt/β-catenin signalling pathway is of great significance to obtain an in-depth understanding of the role of chTERT in ALV-induced tumorigenicity.

In this study, the chTERT gene was amplified from muscle tissue of 4- to 7-day-old chicken embryos. Due to the large size of chTERT, the transfection efficiency of chTERT in chicken-derived cell lines is usually extremely low and not sufficient for research. Therefore, an immortal stable chTERT-overexpressing cell line was constructed by transfection with a lentiviral vector. Before a suitable cell line was selected, chTERT gene expression and telomerase activity in the commonly used chicken-derived cell lines DF-1 and LMH were measured. The results showed that DF-1 cells may not express the chTERT gene and are negative for telomerase, but LMH cells express the chTERT gene at a low level and are positive for telomerase (Figure 1B). Therefore, to conduct further comparative experiments, we infected these two cell lines with recombinant lentivirus and screened for DF-1 and LMH cells stably overexpressing the chTERT gene. However, the expression level of chTERT was significantly lower in DF-1 cells overexpressing chTERT than that in LMH-chTERT cells and decreased rapidly as the cells were passaged until chTERT was no longer expressed (data not shown). DF-1 cells are telomerase-negative immortal cells, not cancer cells, and their immortal proliferation does not depend on chTERT but rather on another mechanism, that is, alternative lengthening of telomeres [42]; this may be why the chTERT gene could not be stably expressed in DF-1 cells. As LMH cells are telomerase-positive cancer cells, the chTERT gene could be stably overexpressed; thus, stable chTERT-overexpressing cells are similar to tumour cells infected with ALV. Therefore, LMH cells were used as the research platform in this study.

This study showed that chTERT can activate the Wnt/β-catenin signalling pathway in LMH cells. Interestingly, after the Wnt/β-catenin signalling pathway was activated by LiCl, the mRNA and protein expression of chTERT and telomerase activity were significantly increased. These data demonstrated that the Wnt/β-catenin signalling pathway can positively regulate chTERT expression. However, whereas the mRNA level of chTERT increased nearly 400-fold in LMH-chTERT cells, the increase in telomerase activity was not as great. First, the TERT mRNA expression level of LMH cells is relatively low, but these cells do have telomerase activity. However, after overexpression of TERT, the mRNA level increased; this is the same thing as having a higher ratio because the denominator is smaller. In this study, telomerase activity was analysed by telomere repeat sequence amplification, a method combining PCR with ELISA. That is, the final results were detected by ELISA after PCR amplification, which led to the change in telomerase activity not being as significant as that in chTERT mRNA expression level. This TRAP method cannot accurately quantify the original telomerase activity but better reflects the trend of change. On the other hand, the obtained results may also stem from a decreased efficiency in translating the foreign HA-labelled TERT gene. The detected protein level of HA-labelled TERT was not as high as expected, and thus, the difference was not as large as that in mRNA levels. This may also explain why the difference in telomerase activity was not as significant as that in the mRNA level. To further confirm that the Wnt/β-catenin signalling pathway can positively regulate chTERT expression, LMH-chTERT cells were transfected with siRNA targeting chTERT. The results were s expected. When the expression of the chTERT gene was inhibited, telomerase activity was reduced, and the activity of the Wnt/β-catenin signalling pathway was significantly decreased. In addition, after the activity of the β-catenin signalling pathway was inhibited by ICG001, IWP-2 and siRNA, the expression of chTERT was significantly reduced, and the activity of telomerase was decreased, further proving the above conclusion. Our results suggested that β-catenin mediates chTERT expression at both the mRNA and protein levels and influences telomerase activity. Moreover, there was a mutually positive regulatory relationship between chTERT and the β-catenin signalling pathway. This finding is consistent with the results of studies on TERT in a variety of human tumours and tumour cells [10, 31, 43].

Many studies have shown that hTERT and β-catenin actively participate in cell growth and development [44, 45] and regulate the development of a variety of human malignancies [46, 47]. To explore whether chTERT and β-catenin play similar roles in LMH cells, we analysed the effect of chTERT on cell growth by CCK-8 assays, RT-PCR, flow cytometry, immunofluorescence staining and TEM. We found that chTERT reduced the number of LMH cells in the G1 phase of the cell cycle and increase the number of cells in S phase and G2; thus, it arrested the cell cycle at S phase, thereby shortening the cell cycle and promoting the proliferation of LMH cells. Moreover, chTERT inhibited the apoptosis of LMH by downregulating the mRNA expression of Caspase 3, Caspase 9 and BAX, which are associated with promoting apoptosis [48]; by upregulating the mRNA expression of BCL-2 and BCL-X, which are associated with inhibiting apoptosis [49]; and by promoting autophagy to inhibit apoptosis.

ALV-J can induce apoptosis and inhibit autophagy to slow cell growth [50–52]. In this study, the overexpression of chTERT in LMH cells inhibited the apoptosis of ALV-J-infected cells and promoted autophagy, ultimately promoting cell proliferation. Therefore, cells overexpressing chTERT can somewhat offset apoptosis induced by ALV-J infection, which indicates that chTERT can improve the viability of cells infected with ALV-J. Thus, compared with control cells, cells overexpressing chTERT significantly promoted the replication of ALV-J. In addition, this study showed that overexpression of chTERT can enhance the migration ability of LMH cells, which confirms that the migratory activity of tumour cells is better than that of normal cells [53]. Finally, we analysed the effect of ALV-J on the expression of chTERT and found that ALV-J significantly increased chTERT mRNA and protein expression. This finding is consistent with previous research showing that chTERT is a common integration site of ALV-J [24].

During the replication process of ALV, the sites of its own genome insertion and integration into the host genes are generally random, which is why tumour formation after infection with chronic transformed ALV is relatively rare. However, our previous study showed that in tumours caused by ALV-J, the virus insertion and integration sites were concentrated in the exon regions of the MYC, TERT and Z1C1 genes or upstream of the transcription sites, preliminarily suggesting that the integration of ALV-J in the host genome is actually weakly selective [24]. In this study, we found that chTERT overexpression promoted the replication of ALV-J, mainly because chTERT promotes virus replication by regulating the Wnt/β-catenin signalling pathway to affect cell growth and provide a better environment for ALV-J replication. On the other hand, when chTERT was overexpressed, the probability of ALV-J insertion and integration near chTERT actually increased. Previous studies have shown that the integration of ALV-J upstream of the chTERT gene can lead to an increase in gene transcription [24]. Therefore, if the probability of ALV-J insertion and integration near chTERT increases, the expression of chTERT will also be upregulated. If the MOI of ALV-J is increased at the same time, then this probability will be further increased; thus, the replication of ALV-J will also affect the expression of chTERT. Increased expression of chTERT in turn promotes cell proliferation and inhibits cell apoptosis, thereby providing a better environment for ALV-J replication. However, stronger evidence is needed to ascertain whether chTERT can directly affect the replication ability of the virus itself.

**Figure 10:**
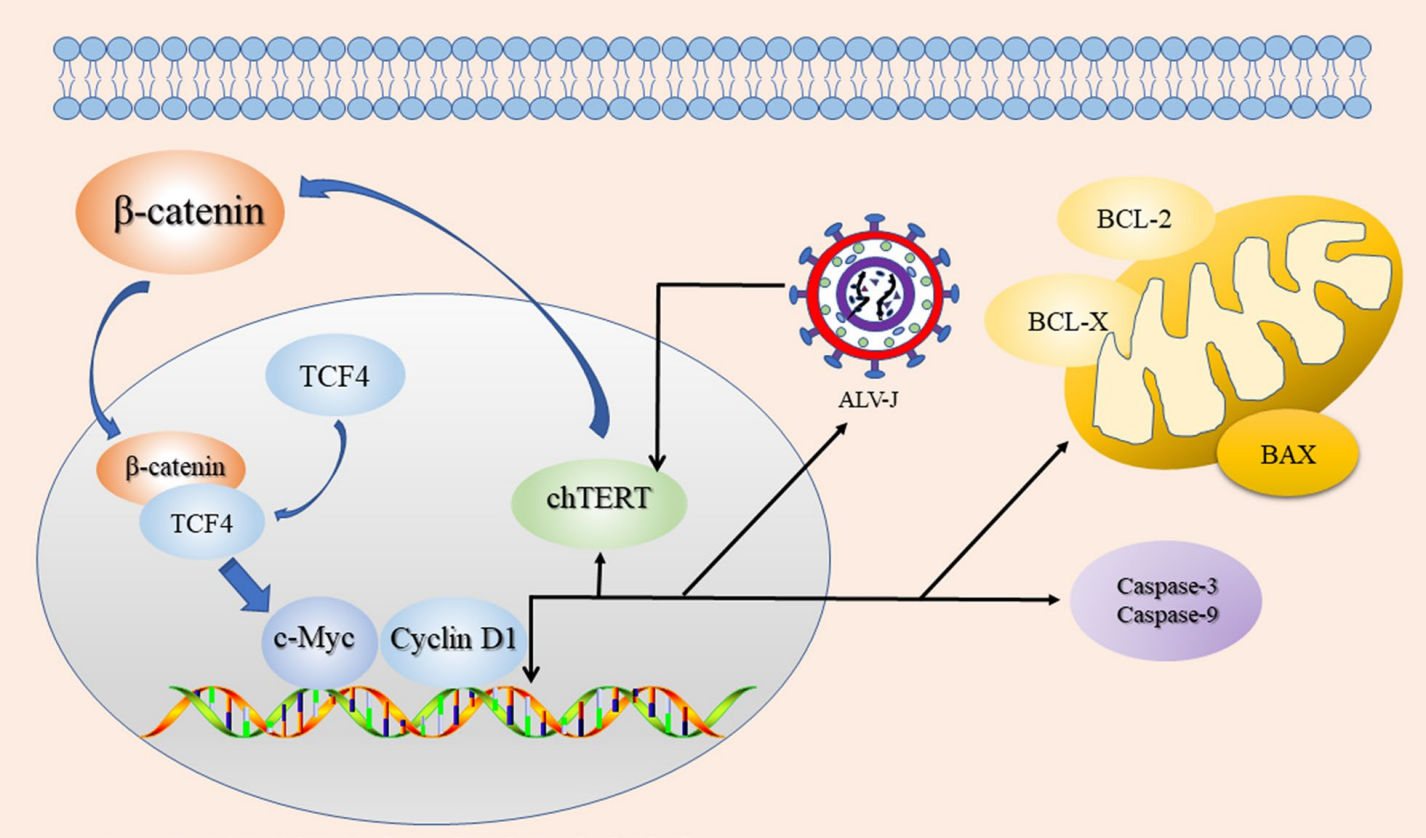
**Schematic diagram of the mutual regulation mechanism between chTERT and the Wnt/β-catenin signalling pathway.**

In summary, chTERT and the Wnt/β-catenin signalling pathway can positively regulate each other in LMH cells, inhibiting apoptosis and promoting autophagy by downregulating the mRNA expression of Caspase 3, Caspase 9 and BAX and upregulating BCL-2 and BCL-X expression. In addition, chTERT can shorten the cell cycle to enhance proliferation and ultimately promote cell migration and the replication of ALV-J in LMH cells (Figure 10). Our findings help reveal the role of chTERT in ALV-J-induced oncogenesis.

## Supplementary Information

**Additional file 1**: **Agarose gel electrophoresis of chTERT gene cloning (A, B and C) and identification of the pLV-chTERT-HA plasmid by restriction enzyme digest (D).**

## Data Availability

The data on which the conclusions of the manuscript rely are presented in the main paper.

## Abbreviations

chTERT: chicken telomerase reverse transcriptase
hTERT: human telomerase reverse transcriptase
ALVs: avian leukosis viruses
ALV-J: avian leukosis virus subgroup J
LMH-chTERT: LMH cells stably overexpressing the chTERT gene
LMH-NC: negative control cells for LMH-chTERT
siRNA: small interfering RNA
TRAP: telomeric repeat amplification protocol
TEM: transmission electron microscopy
ELISA: enzyme-linked immunosorbent assay
MOI: multiplicity of infection
TUNEL: terminal-deoxynucleotidyl transferase-mediated nick end labelling
TCID_50_: 50% Tissue culture infection dose
AA: amino acid
