# Supplementary material for: Mutual regulation between chicken telomerase reverse transcriptase and the Wnt/β-catenin signalling pathway inhibits apoptosis and promotes the replication of ALV-J in LMH cells
Source: Vet Res. 2021 Aug 19;52:110. doi: 10.1186/s13567-021-00979-x (PMC8375160; doi:10.1186/s13567-021-00979-x)

**Additional file 1 Agarose gel electrophoresis**. Cloning of the chTERT gene (A, B and C) and identification of the pLV-chTERT-HA plasmid by restriction enzyme digestion (D).


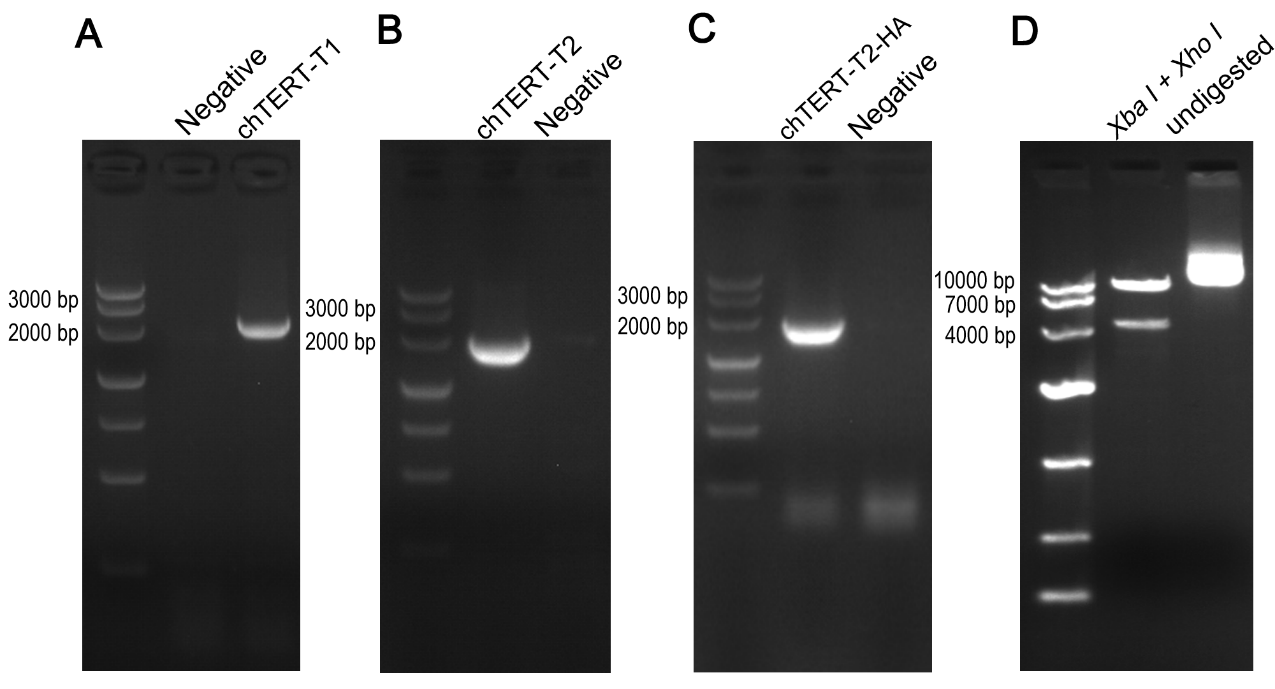

Supplement: Supplementary file 1 — Additional file 1: Agarose gel electrophoresis of chTERT gene cloning (A, B and C) and identification of the pLV-chTERT-HA plasmid by restriction enzyme digest (D). [file 13567_2021_979_MOESM1_ESM.docx]
